# Intrauterine growth restriction and placental angiogenesis

**DOI:** 10.1186/1746-1596-5-24

**Published:** 2010-04-22

**Authors:** Figen Barut, Aykut Barut, Banu Dogan Gun, Nilufer Onak Kandemir, Mehmet Ibrahim Harma, Muge Harma, Erol Aktunc, Sukru Oguz Ozdamar

**Affiliations:** 1 Department of Pathology, Faculty of Medicine, Zonguldak Karaelmas University, Zonguldak, Turkey; 2 Department of Gynecology and Obstetrics, Faculty of Medicine, Zonguldak Karaelmas University, Zonguldak, Turkey; 3 Department of Family Medicine, Faculty of Medicine, Zonguldak Karaelmas University, Zonguldak, Turkey

## Abstract

**Background:**

Vascular endothelial growth factor (VEGF), basic-fibroblast growth factor (b-FGF), and endothelial nitric oxide synthase (eNOS) are factors that take part in placental angiogenesis. They are highly expressed during embryonic and fetal development, especially in the first trimester. In this study, we aimed to investigate the role of placental angiogenesis in the development of intrauterine growth restriction (IUGR) by comparing the levels of expression of VEGF-A, b-FGF, and eNOS in normal-term pregnancy and IUGR placentas.

**Methods:**

The expression of VEGF-A, b-FGF, and eNOS was studied using the avidin-biotin-peroxidase method in placental tissues diagnosed as normal (n = 55) and IUGR (n = 55). Results were evaluated in a semi-quantitative manner.

**Results:**

The expression of all the markers was significantly higher (*p *< 0.001) in cytotrophoblasts, syncytiotrophoblasts, extravillous trophoblasts, vascular smooth muscle cells, chorionic villous stromal cells, and villous vascular endothelial cells of the IUGR placentas when compared with those collected from normal-term pregnancies.

**Conclusion:**

Increased expression of VEGF-A, b-FGF, and eNOS may be the result of inadequate uteroplacental perfusion, supporting the proposal that abnormal angiogenesis plays a role in the pathophysiology of IUGR.

## Introduction

Intrauterine growth restriction (IUGR) is a complicated placental vascular disease resulting in low birth weight, preterm delivery, and increased perinatal morbidity and mortality [1-4]. IUGR may be caused by various fetal, maternal, and placental factors [1-3,5]. Angiogenesis, defined as the development of new vascular structures, is a placental factor playing an important role in the development of IUGR [2-4,6,7].

Angiogenesis involves the branching of new microvessels from pre-existing larger blood vessels. It is an important factor in normal embryogenesis and in physiological processes such as ovulation and the menstrual cycle [8-10]. Angiogenesis plays a role in the development of the villous vasculature and the formation of terminal villi in the human placenta. Placental vascular growth begins early in pregnancy and continues throughout gestation [9,11]. The villous vasculature increases in number rather than vessel type from the 21st day of development until the end of the first trimester. From the 26th week of gestation until term, villous vascular growth changes from branching to non-branching angiogenesis due to the formation of mature intermediate villi that specialize in gas exchange. Specific angiogenic and inhibitory factors regulate these processes [4,8,9]. IUGR occurs as a result of the failure of elongation, branching, and dilation of the capillary loops and of terminal villous formation [4].

Vascular endothelial growth factor (VEGF), basic-fibroblast growth factor (b-FGF), and endothelial nitric oxide synthase (eNOS; type III nitric oxide enzyme) have been identified as positive regulators of angiogenesis [8]. They are strongly expressed during embryonic and fetal development, especially in the first trimester [1,6,10,12-14].

VEGF, one of the first angiogenic factors identified, is widely believed to be the most important regulator of both normal and pathological angiogenesis [15]. It plays an essential role in the formation of new blood vessels [8,12]. In pregnancy, VEGF participates in the proliferation, migration, and metabolic activity of trophoblasts [3,4,6,12]. It is expressed by human villous and extravillous trophoblasts, and conclusive evidence indicates that it regulates trophoblast function by stimulating release of nitric oxide [4]. b-FGF acts as a modulator of tissue differentiation and placental angiogenesis [3,6,10,13], and eNOS has an important role in the regulation of placental blood flow [14].

In this study, we investigated the relationship between placental angiogenesis and the expression of VEGF-A, b-FGF, and eNOS in normal-term pregnancy and IUGR placentas.

## Methods

### Patients

A total of 110 placental tissues from uncomplicated and IUGR pregnancies were included in the study. These were collected from the Zonguldak Karaelmas University Hospital, Department of Gynecology and Obstetrics. Placentas from twin pregnancies, infants with congenital anomalies, fetal aneuploidy, those with proven intrauterine infections, and those from pregnancies with maternal complications such as chronic hypertension, diabetes mellitus, or autoimmune diseases were excluded from the study.

### Tissue samples

Placental tissues were divided in two study groups. The control group consisted of placental tissues collected from 55 women with uncomplicated pregnancies who delivered in the third trimester. The IUGR group contained tissues collected from 55 women in whom IUGR was defined on the basis of an estimated fetal weight of less than the tenth percentile for gestational age, reduced amniotic fluid volume, and Doppler ultrasound of the umbilical artery demonstrating absent end diastolic flow velocity [16]. The diagnosis of IUGR was established by serial obstetric ultrasonographic (ultrasound equipment: GE Logiq 7^®^, Penta Electronics, Ankara, Turkey) examination of fetal measurements such as weight, biparietal diameter, head circumference, femur length, and abdominal circumference. The placentas were weighed after removal of the cords and membranes. Multiple random samples were taken from each placenta, including one from the cord and one from the membrane roll.

### Immunohistochemistry

Expression of VEGF-A, b-FGF, and eNOS was analyzed in 110 placental villous tissues. Samples (1.5 × 1.5 × 1 cm in diameter) taken from the maternal surface of each placenta; infarct areas were excluded from the study. All tissues were fixed in formalin, embedded in paraffin, and cut into 5-μm-thick sections, which were collected on slides coated with poly-L-lysine. After the paraffin was removed, the sections were rehydrated. Immunostaining was performed by the streptavidin-biotin-peroxidase method. Endogenous peroxidase activity was blocked using 3% hydrogen peroxide. Antigen retrieval was carried out in a microwave oven for 15 minutes in 10 nM citrate buffer (pH 6.0) for VEGF-A and eNOS. No antigen retrieval was used for b-FGF antibody. The sections were incubated at room temperature for one hour with RB-222-P rabbit polyclonal antibodies reactive with VEGF-A (a subgroup of VEGF) (1:100; NeoMarkers, Fremont, CA, USA), sc-79 mouse monoclonal antibodies reactive with FGF-2 (147) (1:50; Santa Cruz Biotechnology, Inc., Santa Cruz, California), and rabbit polyclonal antibodies reactive with eNOS (NeoMarkers, Fremont, CA, USA). After washing in phosphate-buffered saline with Tween-20, the tissues were incubated with a biotin-conjugated secondary antibody and then with a biotin-streptavidin complex for 30 min at room temperature. Reactions were visualized with 3,3-diaminobenzidine tetrahydrochloride (DAB). Sections were counterstained with hematoxylin, rinsed, and mounted.

### Evaluation of immunohistochemical staining

The intensity and localization of the staining reaction in chorionic villous stromal cells, vascular smooth muscle cells, villous vascular endothelial cells, cytotrophoblasts, syncytiotrophoblasts, and extravillous trophoblasts was evaluated by two investigators blind to the purpose of the study. Immunoreactivity for antibodies was scored using a semi-quantitative scale for intensity of staining: 0 negative, no staining; 1+ weak positive; 2+ moderately positive; 3+ strongly positive.

### Statistical analysis

Statistical analysis was carried out using SPSS for Windows (version 13.0, Chicago, IL, USA). Continuous variables are presented as mean ± SD. Categorical variables are presented in percentages. Student's *t*-test and Mann-Whitney *U*-test were used to compare groups for clinical and immunohistochemical findings. *P*-values < 0.001 were considered statistically significant.

## Results

Clinical and pathological data, including maternal, neonatal, and placental parameters are shown in Table 1. The mean gestational age of the normal pregnancies and those complicated by IUGR was 38.9 ± 5.9 weeks and 36.3 ± 22.9 weeks, respectively. Birth weight, placental weight, placental diameter, umbilical cord length, and APGAR scores were demonstrated to be significantly different between the two groups.

**Table 1:** Clinicopathological data and statistical comparison between IUGR and normal pregnancy

Clinical data	Normal pregnancy (n: 55)	IUGR (n: 55)	P value
Maternal age (year)	25.9 ± 4.1	27.0 ± 4.5	0.191
Gestational age (week)	38.9 ± 5.9	36.3 ± 22.9	0.000
Apgar score (mean) 1. minute	8.7 ± 1.2	5.9 ± 3.6	0.000
Apgar score (mean) 5. minute	9.7 ± 0.6	7.0 ± 2.9	0.000
Birth weight (g)	3041.5 ± 453.3	2023.9 ± 674.8	0.000
Placental weight (g)	508.9 ± 104.6	404.6 ± 161.2	0.000
Placental diameter (cm)	15.9 ± 1.5	13.6 ± 2.7	0.000
Umbilical cord length (cm)	29.2 ± 4.3	25.6 ± 6.3	0.000

Data were given as mean ± SD

*P: Statistical comparison of normal and IUGR placental tissues

**Figure 1:**
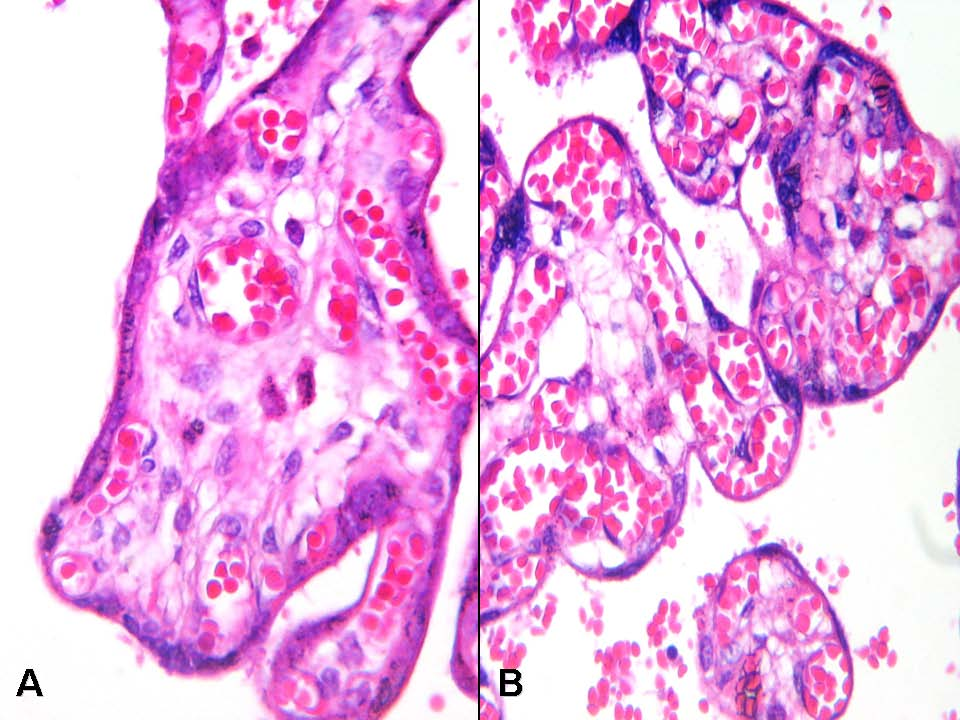
**Histopathologic appearances of placental tissues A; Normal placental tissue B; Increased syncytial knots and villous vascular structures in IUGR placental tissue (H&E A; B; ×200)**.

The clinical and morphological features commonly associated with IUGR observed in 55 placentas are recorded in Table 2. A histological comparison of normal term pregnancy and IUGR placental tissues showed widespread infarct areas and increased syncytial knots or Tenney-Parker changes in the IUGR placentas (Fig. 1A, B).

**Table 2:** The evaluation of clinical and placental datas of 55 pregnancies complicated with IUGR

Clinical data	n (%)	Placental data	n (%)
Cesarean section rate	46 (83.6)	Umbilical cord knots	8 (14.5)
Fetal distress	9 (16.4)	Single umbilical artery	1 (1.8)
Premature rupture of the membranes	2 (3.6)	Placental infarction	51 (92.7)
Preterm birth	4 (7.3)	Placental calcification	31 (56.4)
Prematurity	1 (1.8)	Placental chorangiosis	3 (5.5)
Neonatal intensive care need	13 (23.6)	Chorioamnionitis	4 (7.3)
Neonatal hypoglycemia	1 (1.8)	Accessory lobe	1 (1.8)
Intrauterine fetal death	3 (5.5)		

Staining after incubation with primary antibodies reactive with VEGF-A, b-FGF, and eNOS was observed in the cytoplasm of placental cells from normal term pregnancy and IUGR tissues. Expression of VEGF-A, b-FGF, and eNOS in placental villous tissues was semi-quantified (Tables 3, 4 and 5). A statistically significant (*p *< 0.001) increase in the expression of VEGF-A, b-FGF, and eNOS in IUGR placentas was observed in cytotrophoblast, syncytiotrophoblast, extravillous trophoblast, vascular smooth muscle, and villous stromal and endothelial cells compared with normal term pregnancy placentas.

**Table 3:** Localization and immunostaining intensity of VEGF expression in placental villous tissues

	Normal pregnancy (n: 55)				IUGR (n: 55)				Statistical comparison P value
**Score**	**0** n (%)	**1+** n (%)	**2+** n (%)	**3+** n (%)	**0** n (%)	**1+** n (%)	**2+** n (%)	**3+** n (%)	P*

**CVSC**	-	55 (100)	-	-	-	-	-	55 (100)	<0.001
**VSMC**	-	55 (100)	-	-	-	-	-	55 (100)	<0.001
**VVEC**	-	55 (100)	-	-	-	-	-	55 (100)	<0.001
**ST**	-	55 (100)	-	-	-	-	-	55 (100)	<0.001
**CT**	-	55 (100)	-	-	-	-	-	55 (100)	<0.001
**EVT**		55 (100)	-	-	-	-	-	55 (100)	<0.001

IUGR: Intrauterine growth restricted; CVSC: Chorionic villous stromal cells; VSMC: Vascular smooth muscle cells; VVEC: Villous vascular endothelial cells; ST: Syncytiotrophoblasts; CT; Cytotrophoblasts; EVT: Extravillous trophoblasts [Staining intensity of VEGF were scored as follows: 0 (negative), weak (1+), moderate (2+), and strong (3+)] *P: Statistical comparison of normal and IUGR placental tissues

**Table 4:** b-FGF expression in placental villous tissues

	Normal pregnancy (n: 55)				IUGR (n: 55)				Statistical comparison P value
**Score**	**0** n (%)	**1+** n (%)	**2+** n (%)	**3+** n (%)	**0** n (%)	**1+** n (%)	**2+** n (%)	**3+** n (%)	P*

**CVSC**	-	55 (100)	-	-	-	-	-	55 (100)	<0.001
**VSMC**	43 (78.2)	12 (21.8)	-	-	-	-	-	55 (100)	<0.001
**VVEC**	43 (78.2)	12 (21.8)	-	-	-	-	-	55 (100)	<0.001
**ST**	-	55 (100)	-	-	-	-	-	55 (100)	<0.001
**CT**	-	55 (100)	-	-	-	-	-	55 (100)	<0.001
**EVT**	55 (100)	-	-	-	-	-	-	55 (100)	<0.001

IUGR: Intrauterine growth restricted; CVSC: Chorionic villous stromal cells; VSMC: Vascular smooth muscle cells; VVEC: Villous vascular endothelial cells; ST: Syncytiotrophoblasts; CT; Cytotrophoblasts; EVT: Extravillous trophoblasts [Staining intensity of FGF-b were scored as follows: 0 (negative), weak (1+), moderate (2+), and strong (3+)] *P: Statistical comparison of normal and IUGR placental tissues

**Table 5:** Localization and immunostaining intensity of eNOS expression in placental villous tissues

	Normal pregnancy (n: 55)				IUGR (n: 55)				Statistical comparison P value
**Score**	**0** n (%)	**1+** n (%)	**2+** n (%)	**3+** n (%)	**0** n (%)	**1+** n (%)	**2+** n (%)	**3+** n (%)	P*

**CVSC**	-	55 (100)	-	-	-	-	10 (18.2)	45 (81.8)	<0.001
**VSMC**	48 (87.3)	7 (12.7)	-	-	-	-	14 (25.5)	41 (74.5)	<0.001
**VVEC**	48 (87.3)	7 (12.7)	-	-	-	-	14 (25.5)	41 (74.5)	<0.001
**ST**	-	55 (100)	-	-	-	-	-	55 (100)	<0.001
**CT**	-	55 (100)	-	-	-	-	-	55 (100)	<0.001
**EVT**	55 (100)	-	-	-	-	-	-	55 (100)	<0.001

IUGR: Intrauterine growth restricted; CVSC: Chorionic villous stromal cells; VSMC: Vascular smooth muscle cells; VVEC: Villous vascular endothelial cells; ST: Syncytiotrophoblasts; CT; Cytotrophoblasts; EVT: Extravillous trophoblasts [Staining intensity of eNOS were scored as follows: 0 (negative), weak (1+), moderate (2+), and strong (3+)] *P: Statistical comparison of normal and IUGR placental tissues

**Figure 2:**
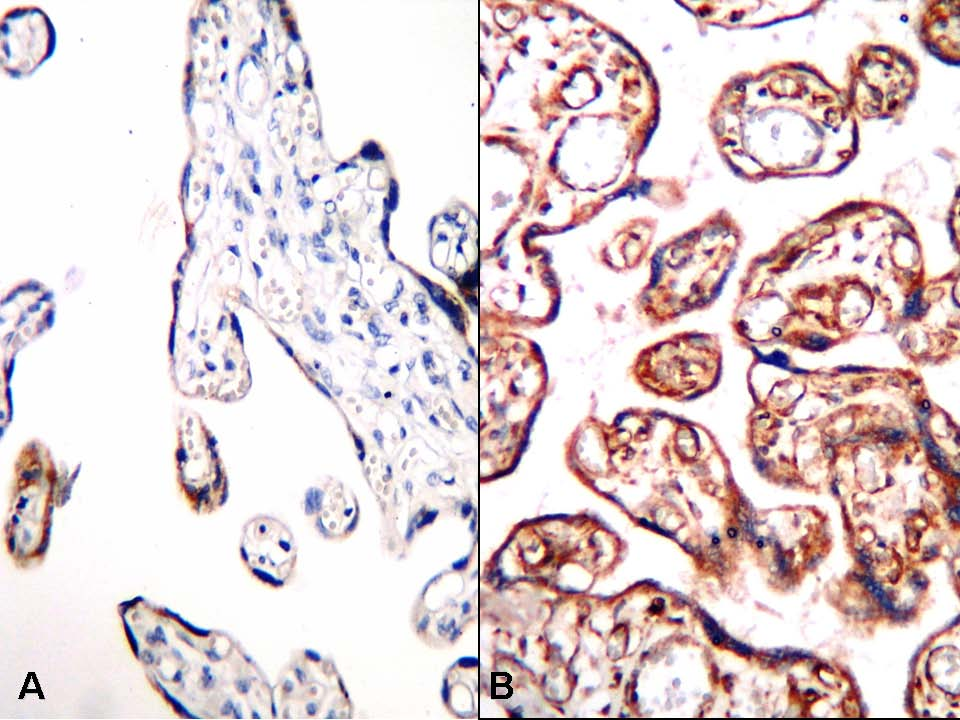
**VEGF expression in placental tissues, A; Weak VEGF expression in cytotrophoblasts and syncytiotrophoblasts in normal pregnancy placenta, B; Strong immune reaction with VEGF in IUGR placental tissue (B-SA peroxidase, DAB, A; B; ×200)**.

**Figure 3:**
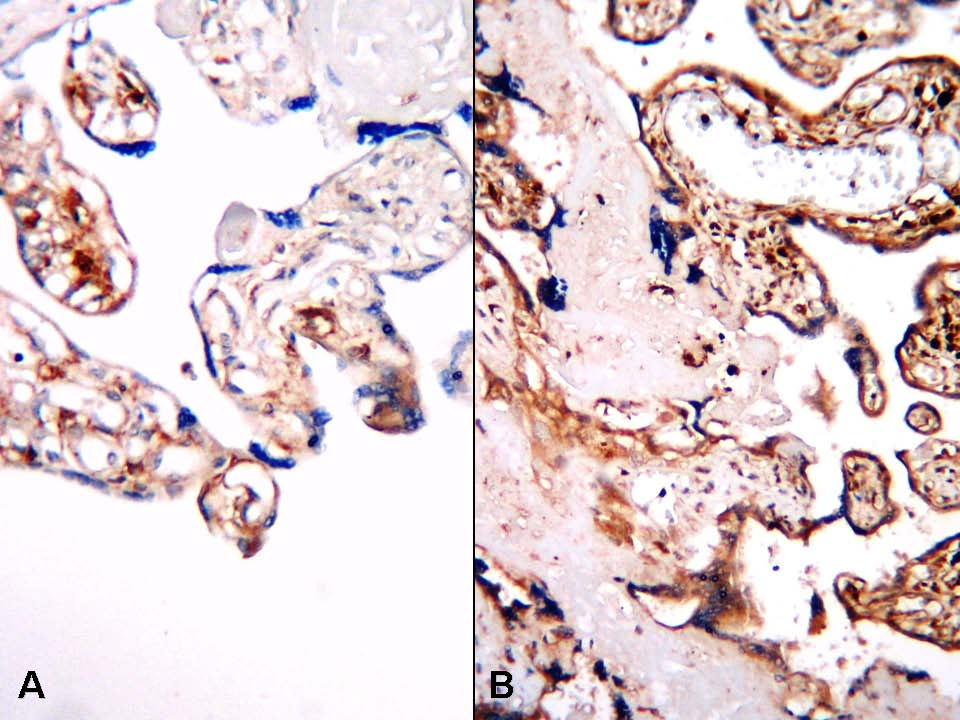
**b-FGF expression in placental tissues, A; Normal placental villous displaying weak b-FGF immune reaction, B; Strong b-FGF expression in IUGR placenta (B-SA peroxidase, DAB, A; B; ×200)**.

**Figure 4:**
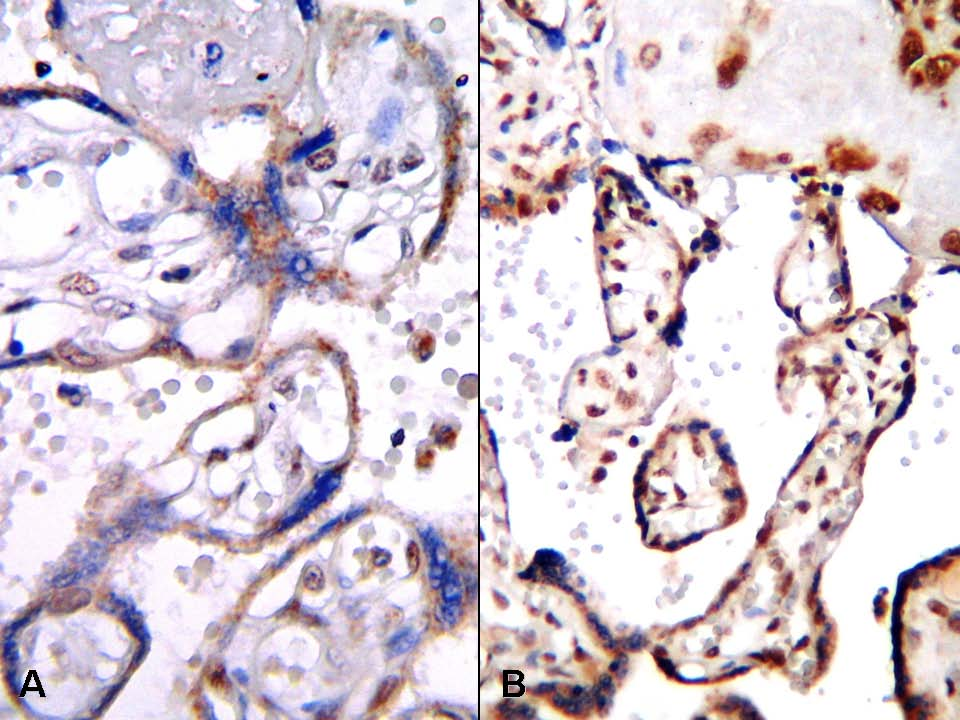
**eNOS expression in placental tissues, A; Weak eNOS expression in normal pregnancy placenta, B; Strong immune reaction with eNOS in IUGR placenta (B-SA peroxidase, DAB, A; B; ×200)**. The English in this document has been checked by at least two professional editors, both native speakers of English. For a certificate, please see: http://www.textcheck.com/certificate/muDdOz

In normal term pregnancy placentas, staining was weak and located predominantly in the cytotrophoblasts and syncytiotrophoblasts (Fig. 2A, 3A, 4A). In IUGR placentas, strong staining was obtained with VEGF-A (Fig. 2B), b-FGF (Fig. 3B), and eNOS (Fig. 4B) primary antibodies.

## Discussion

IUGR is an important cause of perinatal morbidity and mortality. In developed countries, the incidence of IUGR is 3%, whereas in developing countries, it reaches 15-20%. It is one of the most commonly recognized abnormalities of the fetal condition and is a compounding factor in 26% or more of stillbirths [1,4,5]. It may also have long-term health implications for adults [17]. For these reasons, the accurate prediction, diagnosis, and appropriate management of pregnancies complicated with IUGR are important. In accordance with the clinical definition of IUGR, we observed significantly reduced fetal birth weight, amniotic fluid, and placental size and weight in our IUGR group.

The placenta, which normally has a rich vasculature, plays an important role in the development of IUGR. The most common cause of IUGR is placenta ischemia in which insufficient placental function results from deteriorated uteroplacental perfusion [3,5,7,18]. The clinical features of ischemic placental disease are revealed throughout the second half of pregnancy, but the pathophysiological processes initiating the disease originate in the first half [17]. The process of promoting the development of neovessels may be activated by chemo-cytokines in some pathological situations such as ischemia [12]. Structural evidence suggests that placental oxygenation is important in controlling fetoplacental angiogenesis and hence, villous differentiation [9]. Insufficient uteroplacental perfusion leading to abnormal angiogenesis may result in the pathophysiology of IUGR [3,4].

Changes appearing during placental development can be used as early markers of pathologies that may occur later in pregnancy. Placental ischemia is one such pathology, and it can be investigated in relation to various angiogenic mediators [17]. A hypoxic stimulus may lead to an excessive proliferation of villous capillaries and connective tissue via growth factors such as VEGF and FGF [19]. Abnormal vasculogenesis, angiogenesis, and pseudovasculogenesis is correlated with the impaired placental and fetal development seen in complicated pregnancies such as IUGR [3] Angiogenesis may be regulated by oxygen status and by the production of angiogenic growth factors and their natural receptors and antagonists by vascular endothelial cells, pericytes, and trophoblasts. The resulting changes in fetal vasculature are associated with altered patterns of villous growth [18]. The results from our study support the literature reporting that an alteration in placental development accompanying deteriorated angiogenesis occurs in IUGR [4,11,17,18]. Contrary to our findings, Lyall et al. [20] demonstrated a reduction in villous placenta VEGF expression in placental villous tissue from pregnancies complicated by IUGR and preeclampsia.

It is thought that the angiogenic factors VEGF, b-FGF, and eNOS have important roles in villous proliferation, trophoblast function, and angiogenesis as characterized by the formation of new vascular structures in the villous stroma [14]. VEGF is thought to exert a dual role in the placenta, acting on both angiogenesis and trophoblast function during placental development. The roles of VEGF, b-FGF and eNOS in placental angiogenesis may be altered in conditions such as IUGR [4,14]. The increased expression of VEGF-A, b-FGF, and eNOS that we have found in IUGR placentas may promote increased endothelial cell proliferation and migration and pathological angiogenesis [17]. It is likely that normal placental angiogenesis depends on the regulation of vascular development by a complex relationships among these factors and that they play an important role in the development of IUGR.

## Conclusion

It is necessary to explain the regulatory mechanism of placental vascular development in order to elucidate the pathogenesis of IUGR and the associated placental vascular insufficiency. The observed, increased expression of VEGF-A, b-FGF, and eNOS suggests that abnormal angiogenic activity, caused by insufficient uteroplacental perfusion, results in the pathophysiology of IUGR.

## Abbreviations

IUGR: Intrauterine growth restriction; VEGF: Vascular endothelial growth factor; VEGF-A: Vascular endothelial growth factor-A; b-FGF: Basic-fibroblast growth factor; eNOS: Endothelial nitric oxide synthase; H&E: Hematoxylin and eosin; B-SA: Biotin-streptavidin; DAB: 3,3-diaminobenzidine tetrahydrochloride; CVSC: Chorionic villous stromal cells; VSMC: Vascular smooth muscle cells; VVEC: Villous vascular endothelial cells; CT: Cytotrophoblasts; ST: Syncytiotrophoblasts; EVT: Extra villous trophoblasts.

## Competing interests

The authors declare that they have no competing interests.

## Authors' contributions

FB conducted the design of the study, performed microscopic evaluation, and drafted the manuscript. AB participated in the design of the study and performed the selection of appropriate cases and data collection and helped to draft the manuscript. BDG and NOK participated in the design of the study and immunohistochemical evaluation. MIH and MH and SOO conceived of the study, and participated in its design and coordination and helped to draft the manuscript. EA participated in the design of the study and performed statistical analysis. All authors read and approved the final manuscript.
